# Cost-effective surveillance of invasive species using info-gap theory

**DOI:** 10.1038/s41598-021-02299-8

**Published:** 2021-11-24

**Authors:** Yang Liu, Penghao Wang, Melissa L. Thomas, Dan Zheng, Simon J. McKirdy

**Affiliations:** 1 Qingdao Agricultural University, Shandong, 266109 People’s Republic of China; 2 Harry Butler Institute, Murdoch University, Perth, WA 6150 Australia; 3 Food Futures Institute, Murdoch University, Perth, WA 6150 Australia

**Keywords:** Invasive species, Ecological modelling

## Abstract

Invasive species can lead to community-level damage to the invaded ecosystem and extinction of native species. Most surveillance systems for the detection of invasive species are developed based on expert assessment, inherently coming with a level of uncertainty. In this research, info-gap decision theory (IGDT) is applied to model and manage such uncertainty. Surveillance of the Asian House Gecko, *Hemidactylus frenatus* Duméril and Bibron, 1836 on Barrow Island, is used as a case study. Our research provides a novel method for applying IGDT to determine the population threshold ($K$) so that the decision can be robust to the deep uncertainty present in model parameters. We further robust-optimize surveillance costs rather than minimize surveillance costs. We demonstrate that increasing the population threshold for detection increases both robustness to the errors in the model parameter estimates, and opportuneness to lower surveillance costs than the accepted maximum budget. This paper provides guidance for decision makers to balance robustness and required surveillance expenditure. IGDT offers a novel method to model and manage the uncertainty prevalent in biodiversity conservation practices and modelling. The method outlined here can be used to design robust surveillance systems for invasive species in a wider context, and to better tackle uncertainty in protection of biodiversity and native species in a cost-effective manner.

## Introduction

Invasive species can contribute to the extinction of native species, decrease species diversity, and cause considerable ecological and economic loss^1^. Following initial introduction, invasive species have a higher likelihood of being eradicated when detected early with a subsequent rapid response^2^. However, early detection requires effective surveillance to detect small numbers of individuals^3^, often with limited resources. Surveillance programmes have previously been optimized for multiple purposes, for example to minimize the expected time before initial detection or the expected mitigation costs^4^ and minimize the expected total cost of surveillance and incursion management cost^5^. In this paper, we only consider surveillance for detecting the initial incursion of an invasive species. We do not consider delimiting surveillance (to determine the extent of infestation (e.g.^6^)) or monitoring surveillance (to confirm eradication success (e.g. ^7,8^)).

Info-gap decision theory (IGDT) is a non-probabilistic theory that has been used in a range of areas (e.g. engineering model-testing, financial risk assessment and marine protection policy decisions^9^). Such robust decision making methods are often desirable in ecological systems characterized by Knightian uncertainty^10^. Knightian uncertainty is characterized by high knowledge-deficiency of the probability or frequency of outcomes, as opposed to the risk that can be measured probabilistically^11^. Various methods to support decisions in face of Knightian uncertainty have been developed^12,13^. Wald’s maximin to ameliorate worst outcome has been used as one of the primary methods. However, it is also known for excessive conservatism and pessimistic^14^. IGDT offers an alternative of Wald’s maximin to quantify the confidence in realising specified aspirations but enable a balance between them as a robust-satisfying method^15^.

Knightian uncertainty may exist in parameter estimates, regarded as parameter uncertainty. IGDT has been used previously to manage invasive species (e.g.^16–18^), however, this project is the first application of IGDT in determining the population threshold ($K$) for detection of an invasive species. The population threshold ($K$) is the population size for which the surveillance programme is designed to detect at least one individual of invasive species. Given that the population threshold of a species is based on the risk tolerance of managers and the biology of the species in question, it is difficult to provide actual values against this parameter. Despite this, such management parameters are widely used (e.g.^19,20^). Because of the direct relation between surveillance cost and population size^21^, these population threshold estimates could have significant cost consequences if underestimated.

Knightian uncertainty may also exist in uncertainty models and in system models (i.e. reward function), known as structural or functional uncertainty (e.g.^22^). In the case of surveillance cost and population threshold, how these two factors interact is likely to be different for each species or for various surveillance period design, e.g. long-term spatial-dynamic models^23^ and static optimisation methods over one-time period^24^. Using a different method for calculating surveillance cost could potentially change the population threshold determined.

IGDT is a method that not only comprises economic deliberation, but also quantitatively represents Knightian uncertainty where there is no objective measure of probability^15^. In our research, IGDT is applied to quantitatively represent the maximum Knightian uncertainty against which robust decisions with satisfactory performance are generated. In this study, we use the potential introduction of the Asian House Gecko, *Hemidactylus frenatus* (AHG), onto Barrow Island (BWI), as a case study.

The Asian House Gecko is an excellent hitchhiker and has expanded its range due to increased vessel and goods movement^25^. AHG is of increasing concern for its potential effects on the ecosystem. This species is known to carry novel parasites, out-compete other native gecko species and predate on invertebrates and geckos^26^. It is considered a high-risk species for incursion onto BWI and is known to be difficult to detect once introduced^27^. BWI was set aside as a reserve by Western Australia legislature in 1910 due to its conservation value^17^. The Gorgon Project commenced construction of a liquefied natural gas processing plant on BWI in 2009^28^. A condition of the Gorgon Project approval was inclusion of a non-indigenous species surveillance program that could detect an introduced species (with a statistical power of detection of 0.8 or greater) if the species was present on the island due to Gorgon Project activities^29^. To meet this ministerial requirement, a surveillance system model was developed^30^. This model is primarily reliant on expert elicitation for input values, which may underestimate the uncertainty in the model parameters with lack of data and other potential and subtle independent influences^31^, and result in exceeding budget limits.

This research was conducted to determine the population threshold value that allows the uncertain model parameter values to depart as much as possible from their estimated values while satisfying budget constraint. This could help decision makers to make robust-optimal decision that maximize the immunity against detection failure while meeting cost constraints. Our study provides a new method for designing robust surveillance, contributing to efficient application of limited resources and reducing financial and environmental costs.

## Methods

### Data

The Surveillance System for terrestrial vertebrates on BWI has undergone a large number of reviews (e.g.^17^) since the commencement of the Gorgon Project. The reviews take into consideration the operations being undertaken at the time. These include changes to the volume of cargo, number of personnel travelling to BWI and the ports of origin. Our study is based on revised input data from the most recent studies (e.g.^32^).

**Figure 1:**
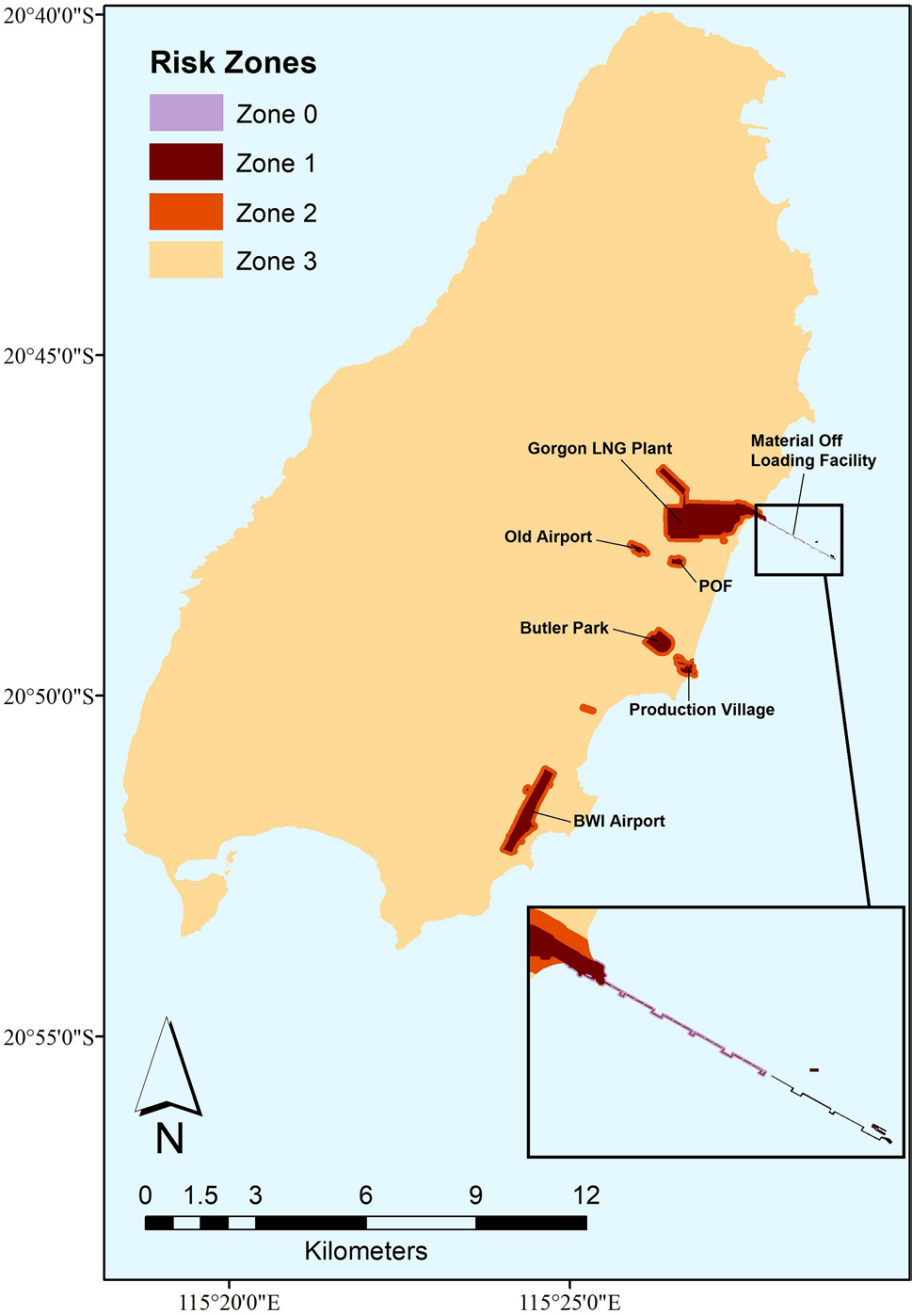
Quarantine invasion risk map of the Asian House Gecko (AHG) on Barrow Island. Invasion risk was calculated by multiplying relative importance weights (RIWs) for entry points (see Table S1) and RIWs for establishment (see Table S2), and categorized into four ranges for mapping as described by Jarrad et al.^30^. 100 m was added around all entry points as the buffering to indicate the potential home range of AHG. The risk map was generated using the sf (1.0.1), ggmap (3.0.0), and ggplot2 (3.3.3) packages in R (version number 4.0.4, URL https://www.R-project.org) from shapefiles provided by Chevron. Zone 1 is the area with the highest occupancy probability, where the majority of the surveillance budget should be spent. This zone consists of areas where high risk activities for incursion are undertaken. These activities include the unloading of cargo (laydown areas), the receipt of planes (airports) and marine vessels (marine ports). Zone 2 is the secondary introduction area (100 m buffer area around Zone 1) and is considered the lower risk boundary for a species dispersing out of Zone 1. Zone 0 is the buffer area at Material Offloading Facility (MOF) (i.e. X-Blocs area). This is because the habitat at Zone 2 at the MOF is highly different to that at all other locations, making the characters of Surveillance System Components (SSCs) in Zone 2 at the MOF (i.e. X-Blocs) different from that in Zone 2 at other locations. Zone 3 is the remaining island area where the AHG is less likely to establish prior to detection thus with no SSCs allocated.

Our model considers six primary or secondary points of entry (Supplementary Table S1, Fig. 1). In addition, three preferred habitat types were the focus of island surveillance for AHG (Supplementary Table S2), as opposed to surveying the entire island. AHG are rarely found in natural areas in their introduced range and prefer anthropologically modified areas, particularly where there is artificial lighting that attracts insect prey^33^.

For this model, BWI has been spatially separated into four zones (See Fig. 1 for more details). AHG are excellent climbers, and are regularly found on walls, under buildings and on ceilings^34^. For this reason, the area for surveillance in Zone 1 is focused on and around buildings and was calculated based on a three-dimensional searching area. The surveillance area of each location is shown in Supplementary Table S3. There are six Surveillance System Components (SSCs) ($i = 1,2...,6$) used to detect the AHG on BWI (Supplementary Table S4). The characters of these SSCs at different locations and zones are summarized in Supplementary Table S5. The surveillance period designed for this model is one year since the probability of AHG being detected changes due to seasonal elements within a year^35^.

### Model methods

Info-gap analysis requires three primary components, each of which builds on the last; a system model, the performance requirement and the uncertainty model^36^. Following Jarrad et al.^30^ and Whittle et al.^35^, the quantity of SSC $i$ to be used in each zone $j$,$N_{i}^{j}$ ($N_{i}^{j} \ge 0$), could be calculated as Eq. (1):

1 $$ N_{i}^{j} = \frac{{\log (\mathop \beta \nolimits^{{\frac{1}{{(1 - \sigma_{i}^{j} F_{i}^{j} )C_{i} ({1 \mathord{\left/ {\vphantom {1 {R^{j} }}} \right. \kern-\nulldelimiterspace} {R^{j} }})}}}} )}}{{K\log (1 - \sigma_{i}^{j} F_{i}^{j} )}}(K \ge 0,\sigma_{i}^{j} \le 1,F_{i}^{j} \le 1,R^{j} \le 1,C_{i} > 0) $$

where $i$ is the type of SSC (Supplementary Table S4); $j$ is the risk zone; $R^{j}$ indicates the probability of occupancy in risk zone $j$; $\beta$ is the probability of a type 2 error (the probability of falsely declaring the invasive species to be absent); $\sigma_{i}^{j}$ is the detection probability of SSC $i$ in zone $j$ given invasive species present in the footprint (footprint is the area in which an AHG can be detected with a single unit of SSC); $F_{i}^{j}$ is the sampling fraction covered by one SSC unit of $i$ in zone $j$ (i.e. footprint/total target area); $K$ is the population threshold for detection; $C_{i}$ is the cost per unit SSC $i$.

Therefore, the system model in this research, i.e. the total surveillance cost used at all locations, is $r = \sum\nolimits_{i} {\{ (\sum\nolimits_{L} {(\sum\nolimits_{j} {{\text{ceil}}(N_{i}^{j} } \times \frac{{A_{L}^{j} }}{{A_{t}^{j} }})} } )) \times C_{i}  \}$, where $A_{L}^{j}$ is the area of zone $j$ at location $L$, $A_{t}^{j}$ is the total area of zone $j$ (see Supplementary Section S1 for details).

The occupancy probabilities ($R^{j}$) of AHG in Zone 0, 1 and 2 during the early stage of invasion is set to 0.1, 0.8 and 0.1 respectively, based on subject matter expert input. To reach Gorgon’s regulatory requirement of a specified detection power of 0.8, $\beta$ is set to 0.2. Wintle and Burgman^32^ estimate the population threshold for AHG to be eight for the whole island ($\tilde{K} = 8$), based on subject matter expert elicitation and actual data from the eradication of AHG from BWI in 2015, where seven AHGs were detected and eradicated. The value of other parameter estimates have been given by independent vertebrate specialists either through expert elicitation or surveying (Supplementary Table S5). The circumstances in which $K,\sigma ,F,R$ are uncertain will vary from study to study. For example, in circumstances where the footprint of all SSCs for AHG can be measured precisely, there exists no uncertainty in the value of $F$.

Assume that the total surveillance cost is not able to exceed $r_{c}$, in other words, a budget limit of $r_{c}$ is proposed. It is highly desirable that the total surveillance cost does not exceed the lower value $r_{w}$, $r_{w} \le r_{c}$.

We consider 40 different uncertain parameters: $K,\sigma_{i}^{j} ,F_{i}^{j}$ and $R^{j}$ for $i = 1,...,6$ and $j = 0,1,2$ . We will refer generically to these 40 uncertain parameters with the vector $x = (x_{1} ,...,x_{40} )$ but refer explicitly to $K,\sigma_{i}^{j} ,F_{i}^{j}$ and $R^{j}$ for clarity or simplicity. We have initial parameter estimates denoted by the known 40-vector $\tilde{x}$. The only additional information available is that the parameters are all non-negative, and all parameters other than $K$ are bounded above by one. We have no further information. For instance, probability distributions for uncertainty in these parameters are lacking. In face of this severe uncertainty we will use fractional-error info-gap model of uncertainty (Eq. (2)). Analogous to deviations around the mean in Bayesian approach, info-gap uncertainty model (Eq. (2)) measures the fractional deviation between the parameters and the estimates, but without probabilities assigned^15^.

2 $$ U(h) = \{ x:|\frac{{x_{n} - \tilde{x}_{n} }}{{\tilde{x}_{n} }}| \le h,x_{n} \ge 0,n = 1:40;\sigma_{i}^{j} \le 1,F_{i}^{j} \le 1,R^{j} \le 1,i = 1:6,j = 0:2\} ,h \ge 0 $$

Robustness can be mathematically explained as the greatest horizon of uncertainty (i.e. the maximum of a set of values of $h$ in Eq. (2)) up to which the system model obeys the performance requirement, $r \le r_{c}$. It measures the confidence in realising the desired goals facing Knightian uncertainty. The robustness function is defined as $\hat{h}(r_{c} ) = \max \{ h:(\mathop {\max }\limits_{K,\sigma ,F,R \in U(h)} r) \le r_{c} \}$. Let $m(h)$ denote the inner maximum ($m(h) = \max r$) and $m(h)$ is the inverse function of $\hat{h}(r_{c} )$ (see Supplementary Section S1 for details).

Opportuneness is the least level of uncertainty that has to be tolerated to facilitate windfall outcomes as wonderfully small as $r_{w}$. The opportuneness function in this model is $\hat{\beta }(r_{w} ) = \min \{ h:(\mathop {\min }\limits_{K,\sigma ,F,R \in U(h)} r) \le r_{w} \}$. Let $w(h)$ denote the inner minimum ($w(h) = \min r$) and $w(h)$ is the inverse function of $\hat{\beta }(r_{w} )$ (see Supplementary Section S2 for details).

All the exploratory analyses of robustness and opportuneness function have been conducted in MATLAB R2018b^37^.

## Results

**Figure 2:**
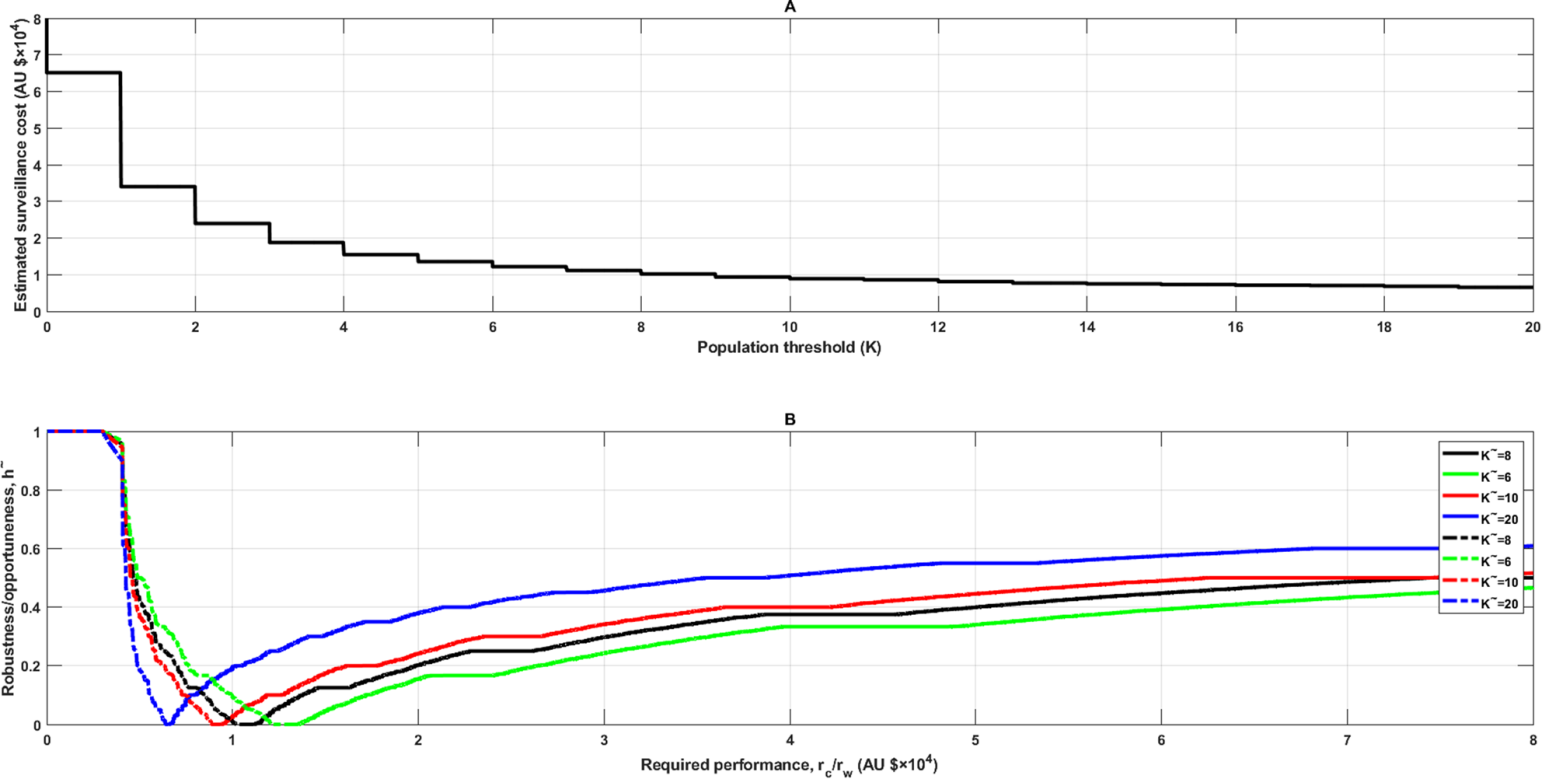
**(A)** Relationship between population threshold ($K$) of Asian House Gecko and estimated surveillance cost based on the initial estimates of uncertainty parameters $\sigma ,F,R$ (Drawn according to Supplementary equation (S3)); **(B)** robustness/opportuneness curves when $K,\sigma ,F,R$ are uncertain using the initial estimates $\tilde{\sigma },\tilde{F},\tilde{R}$ and $\tilde{K} = 6,8,10,20$ respectively. Solid lines are robustness curves and dashed lines are opportuneness curves (online version in colour).

The predicted surveillance cost based on the initial estimates of uncertainty parameters $$\sigma ,F,R$$ decreases hyperbolically with an increasing AHG population threshold (Fig. 2A). The estimated surveillance cost with the initial estimate for $$K(\tilde{K} = 8)$$ and $$\sigma_{i}^{j} ,F_{i}^{j} ,R^{j}$$ is AU$11,143, which seems to be a relatively small cost. However, these initial estimates are subjectively based on the information experts had available at the time, and may not be necessarily accurate. To solve this problem, the robustness is taken into consideration.

Figure 2A illustrates that the surveillance cost is very large when $K$ is four or less, but the curve flattens out when it is between six and twenty. This indicates that a $K$ value of between six and twenty would be more economically sustainable. We therefore evaluated the robustness and opportuneness using $K$ estimate values of 6, 8, 10 and 20.

### Robustness curve when $K,\sigma ,F,R$ are uncertain

The increasing solid curves in Fig. 2B are robustness curves. The step-wise continuity of these robustness curves (Fig. 2B) is a result of the discrete values attainable by $N$ and $K$, as expressed by the “ceil” function in Supplementary equation (S12). There are two properties that should be noted, including zeroing and the trade-off property^36^.

The zeroing property affirms that predicted outcomes have zero robustness to uncertainty. Each curve reaches the horizontal axis (for which robustness is zero) precisely when the required performance equals the predicted value (Fig. 2B). For instance, consider the black curve, calculated with an estimated surveillance cost of AU$11,143, for which the robustness equals zero (Fig. 2B). The trade-off property asserts that the robustness improves (increases) as the performance requirement worsens (increases). This is reflected in the robustness curves of Fig. 2B which increase monotonically: the robustness increases as the maximum allowed surveillance cost increases; $$r_{c} < r_{c} ^{\prime}$$ implies $$\hat{h}(r_{c} ) \le \hat{h}(r_{c} ^{\prime})$$ (p46^36^).

Comparing four robustness curves in Fig. 2B, the estimated surveillance cost (when robustness is zero) is lowest on curve ‘$$\tilde{K} = 20$$’. However, this is not reliable because robustness of zero means that there is no immunity to uncertainty. Curve ‘$$\tilde{K} = 20$$’ is preferred to the other three curves because it has higher robustness at the same value of critical budget. For example, the largest error in the estimation of parameters (i.e. robustness), ± 50% (0.50) would guarantee the total surveillance cost to be no more than required AU$38,572 when using ‘$$\tilde{K} = 20$$’.

### Opportuneness curve when $K,\sigma, F, R$ are uncertain

The decreasing dashed curves in Fig. 2B are opportuneness curves. Robustness and opportuneness curves converge on the horizontal axis at the point reaching the predicted surveillance cost, i.e. zeroing property (Fig. 2B). Zeroing for opportuneness means that no uncertainty is needed to enable the expected outcome and a small value of the opportuneness function is desirable. The value of opportuneness decreases along with the required surveillance cost. Opportuneness shows the immunity to windfall success, therefore a smaller value is better than a large value. Opportuneness improves as $r_{w}$ increases, i.e. trade-off property. Opportuneness curve ‘$\tilde{K} = 20$’ is lower than the other three curves (Fig. 2B) and is thus preferred. We see that robustness and opportuneness are sympathetic in this example: Any change in $\tilde{K}$ that improves robustness, also improves opportuneness.

## Discussion

Ecological systems, and the species within, are highly complex and variable. This complexity makes it difficult for model input parameter values to be derived from empirical data or for uncertainty in these parameters to be parameterized probabilistically. This results in Knightian uncertainty underlying ecological analysis and may consequently skew policy outcomes. For example, in our study, in which the performance requirement is no more than a critical budget value of AU$40,000 (Fig. 2B), if any of the parameter estimates (when $$\tilde{K}$$ is eight) err by more than ± 37.5%, the total surveillance cost will be considerably over AU$40,000. To avoid the burden of excessive surveillance costs, it is critical to have a robust approach to prevalent uncertainty, such as that outlined in this research.

Our research shows that the estimated cost for surveillance for a very small population threshold (less than four) of AHGs is large (Fig. 2A). This is because low population densities reduce the probability of detection success, consequently for detection to occur, more resources will be required^21^. An example of this is the annual trapping cost for gypsy moth in North America, which Bogich et al.^38^ showed there was a linear function of trap density and total area, with more traps required when the population size is small^30^. Counter to this, robustness increases as size of the budget becomes larger (Fig. 2B).

An increase in robustness allows for more mistakes in the parameter estimates to be tolerated when making decisions, which is beneficial in invasive species management. One way to improve robustness is by increasing the size of the budget (Fig. 2B). The other is by increasing the population threshold within the possible population threshold range, which simultaneously improves opportuneness. The robustness gained with increasing the population threshold from six to ten is similar to increasing the population threshold from ten to twenty at the same level of required performance (Fig. 2B). How big the population threshold is, is a matter of risk appetite, however, further increase in population threshold is not recommended as this could result in the species becoming well established prior to being detected, and consequently, eradication becoming more difficult and expensive.

For robustness, ‘bigger is better’, however, ‘big is bad’ for opportuneness^36^. A lower value associated with the opportuneness function means less uncertainty has to be tolerated, i.e. higher opportunity to seek lower surveillance cost. In the case of AHG, the scenario in which ‘$\tilde{K} = 20$’ (Fig. 2B) is not only robust-dominant but is also opportuneness-dominant at the same level of required surveillance cost, thus would be preferred by many decision makers. However, consideration should be given to the entire biosecurity continuum when deciding the final surveillance population threshold. This is because a lower population threshold can decrease eradication costs by ensuring populations are detected when they are low in numbers and before they have spread^39^. A population of 20 AHG is likely to be more difficult to eradicate, and have a greater environmental impact, than a population of only eight AHG. The population threshold should therefore be sufficiently high to enable detection without significant surveillance costs, but low enough to enable successful eradication without major impact. IGDT has rarely been applied to assist in decision making of eradication campaigns (e.g.^18^), and the relationship between detection surveillance and eradication (e.g.^38,39^) will be explored in future research using this model.

Our methodology demonstrates how robustness/opportuneness curves may be applied to determine if the size of the budget is ‘sufficiently robust/opportune’. Determining a suitable value of robustness/opportuneness is dependent upon the trade-offs deemed appropriate by decision makers. If the decision-makers aim to reach ambitious goals (i.e. low critical surveillance cost), they may need to compromise on the level of confidence in realizing them^15^. Conversely, if decision-makers ideally require high confidence in realizing specified targets, then the targets could be moderated by ambitiousness (i.e. they may need to improve the critical surveillance cost). Such compromises in invasive species management requirements could be compensated by achieving greater robustness against error in the parameter estimates.

The IGDT-based method explored in this research does not optimize (minimize) the cost spent on surveillance but concentrates on identifying the robust population threshold against the uncertainty existing in $\tilde{K},\tilde{\sigma },\tilde{F},\tilde{R}$ for implementing eradication of a known invasive species, while simultaneously satisfying a cost requirement. The method can easily be tailored to specific requirements associated with varying degrees of uncertainty. As uncertainty parameters may not be restricted to the parameters analysed in this research, more uncertainty parameters could be included, e.g. the surveillance cost-effectiveness, population growth rate, discount rate that may be considered in spatial-dynamic models analysis. On the other hand, if some of the parameters are based on reliable information and a smaller number of parameters are subjected to uncertainty, the model could be simplified and potentially lead to more robust results. As Rout et al.^18^ suggested, the same horizon of uncertainty $h$ can be applied to measure the uncertainty for different parameters ($K,\sigma ,F,R$ in this research). The method gives decision makers freedom in both what and how to tackle uncertainty to allow robust optimal decisions under varying scenarios.

Early detection faces the challenge of reconciling limited budget with comprehensive sampling^40^. Ecological uncertainty, distributional uncertainty and taxonomic uncertainty were recognised as three basic underlying sources of uncertainty that may ultimately affect management decisions^41^. This research offers a flexible application of IGDT that can be applied not only to the detection of invasive species, but also incorporated into detection of rare and endangered species that are inherently hard to find. This method may also be used directly in the robust surveillance design in areas other than biodiversity protection, such as the grain industry and aquaculture. This research could provide a guide to decision makers to determine other appropriate robust thresholds regarding when and which strategy should be implemented for a variety of ecological management purposes.

## Supplementary Information

Supplementary Information.

## Data Availability

Data available via the Dryad Digital Repository https://doi.org/10.5061/dryad.fxpnvx0rp.
