# Supplementary material for: Cost-effective surveillance of invasive species using info-gap theory
Source: Sci Rep. 2021 Nov 24;11:22828. doi: 10.1038/s41598-021-02299-8 (PMC8613277; doi:10.1038/s41598-021-02299-8)
Supplement: Supplementary file 1 — Supplementary Information. [file 41598_2021_2299_MOESM1_ESM.pdf]

Cost-effective surveillance of invasive species using info-gap theory

Yang Liu[1,2]*, Penghao Wang[2,3], Melissa L. Thomas[2], Dan Zheng[1]* and Simon J. McKirdy[2]*

[1]*Qingdao Agricultural University, Shandong 266109, P.R. China*

[2]*Harry Butler Institute, Murdoch University, Perth, WA 6150, Australia*

[3]*Food Futures Institute, Murdoch University, Perth, WA 6150, Australia*

**Corresponding author*

E-mail: Yang.Liu@murdoch.edu.au; zhengdan1818@163.com; s.mckirdy@murdoch.edu.au

Supplementary Information

Section S1: Derivation of the robustness function

The total number of Surveillance System Components (SSCs) to be used should be the sum of SSCs at different locations L depending on the relative area A_L^j / A_t^j (A_L^j is the area of zone j at location L, A_t^j is the total area of zone j). For example, if the gecko scat analysis used in Zone 1 is calculated to be one unit, when this specific SSC is allocated to six different locations in Zone 1, each of the locations should be allocated one unit and thus there will be six units in total for all locations. The number of SSCs applied at six different locations will be rounded up to the next highest value taking the relative area into consideration. Function 'ceil' is used in this

research to round up to the next highest value. For example, for the values 4.2 and 4.8, the final value would be five. Define the quantity of surveillance type i used in zone j is N_i^j, so the quantity of surveillance type i used at location L is:

$$n_i^L = \sum_j \text{ceil}(N_i^j \times \frac{A_L^j}{A_t^j})\ (n_i^L \geq 0, N_i^j \geq 0, A_L^j \geq 0, A_t^j \geq 0) \quad \text{(S1)}$$

where,

$$N_i^j = \frac{\log(\beta^{\frac{1}{(1-\sigma_i^j F_i^j)C_i(1/R^j)}})}{K\log(1-\sigma_i^j F_i^j)} (K \geq 0, \sigma_i^j \leq 1, F_i^j \leq 1, R^j \leq 1, C_i > 0) \quad \text{(S2)}$$

Therefore, the total surveillance cost on the whole island is:

$$r = \sum_i (\sum_L n_i^L \times C_i) = \sum_i \{(\sum_L (\sum_j \text{ceil}(N_i^j \times \frac{A_L^j}{A_t^j}))) \times C_i\}$$

$$= \sum_i \{(\sum_L (\sum_j \text{ceil}(\frac{\log(\beta^{\frac{1}{(1-\sigma_i^j F_i^j)C_i(1/R^j)}})}{K\log(1-\sigma_i^j F_i^j)} \times \frac{A_L^j}{A_t^j}))) \times C_i\}$$

(S3)

As referred in the body of the paper, we will refer generically to the 40 uncertain parameters (K, σ_i^j, F_i^j and R^j for $i = 1,...,6$ and $j = 0,1,2$) with the vector $x = (x_1,...,x_{40})$ and sometimes refer explicitly to K, σ_i^j, F_i^j and R^j for clarity or simplicity. We also have initial estimates of K, σ_i^j, F_i^j and R^j, denoted by the 40-vector $\tilde{x}$.

The fractional-error info-gap model of uncertainty is defined as:

$$U(h) = \{x : |\frac{x_n - \tilde{x}_n}{\tilde{x}_n}| \leq h, x_n \geq 0, n = 1:40; \sigma_i^j \leq 1, F_i^j \leq 1, R^j \leq 1, i = 1:6, j = 0:2\}, h \geq 0$$

(S4)

Then the info-gap's robustness function is defined as:

$$\hat{h}(r_c) = \max\{h : (\max_{K,\sigma,F,R \in U(h)} r) \leq r_c\} \quad \text{(S5)}$$

where $\hat{h}$ is the greatest horizon of uncertainty up to which the system model obeys the performance requirement.

Let $m(h)$ denote the inner maximum in the definition of the robustness function. The sets of the info-gap model, $U(h)$ become more inclusive as h increases. This implies that $m(h)$ increases as h increases. From the definition of the robustness we see that $\hat{h}(r_c)$ is the greatest value of h at which $m(h) \leq r_c$. This means that a plot of h vs $m(h)$ is equivalent to a plot of $\hat{h}(r_c)$ vs r_c. In other words, $m(h)$ is the inverse function of $\hat{h}(r_c)$. Once we derive an algebraic expression for $m(h)$ we can plot the robustness curve; we do not need to invert $m(h)$, which can be problematic. We now proceed to derive $m(h)$. Plots of robustness curves in the body of the manuscript are based on the expressions for $m(h)$.

Let $m(h)$ denote the inner maximum,

$$m(h) = \max r = \max \sum_i \{(\sum_L (\sum_j \mathrm{ceil}(\frac{\log(\beta^{\frac{1}{(1-\sigma_i^j F_i^j) C_i (1/R^j)}})}{K \log(1 - \sigma_i^j F_i^j)} \times \frac{A_L^j}{A_t^j}))) \times C_i\} \leq r_c \quad \text{(S6)}$$

Since both σ_i^j and F_i^j are less than one, the value of $\log(1-\sigma_i^j F_i^j)$ must be negative. And K is positive, thus the denominator must be negative. $(1-\sigma_i^j F_i^j)\times C_i \times (1/R^j)$ is positive and $\beta = 0.2$, $\beta^{\frac{1}{(1-\sigma_i^j F_i^j)C_i(1/R^j)}} < 1$, thus the value of numerator is negative. In order to get maximum of r, the negative denominator should be as large as possible and negative numerator as small as possible. Therefore, K, σ, F should be minimum and R be maximum.

It is defined here:

$$\xi^+ = \begin{cases} \xi, & 0 \le \xi \le 1 \\ 0, & \xi < 0 \\ 1, & \xi > 1 \end{cases} \quad \text{(S7)}$$

Then

$$\sigma_i^j = (1-h)^+ \tilde{\sigma}_i^j \quad \text{(S8)}$$

$$F_i^j = (1-h)^+ \tilde{F}_i^j \quad \text{(S9)}$$

$$R^j = ((1+h)\tilde{R}^j)^+ \quad \text{(S10)}$$

$$K = (1-h)^+ \tilde{K} \quad \text{(S11)}$$

Therefore,

$$m(h) = \max r = \max \sum_i \{(\sum_L (\sum_j \text{ceil}(N_i^j \times \frac{A_L^j}{A_t^j}))) \times C_i\}$$

$$= \sum_i \{(\sum_L (\sum_j \text{ceil}(\frac{\log(\beta^{\frac{1}{[1-(1-h)^+ \tilde{\sigma}_i^j (1-h)^+ \tilde{F}_i^j] C_i [1/((1+h)\tilde{R}^j)^+]}})}{(1-h)^+ \tilde{K} \log[1-(1-h)^+ \tilde{\sigma}_i^j (1-h)^+ \tilde{F}_i^j]} \times \frac{A_L^j}{A_t^j}))) \times C_i\} \le r_c$$

(S12)

Section S2: Derivation of the opportuneness function

The opportuneness function in this model is defined as:

$$\hat{\beta}(r_w) = \min\{h : (\min_{K,\sigma,F,R \in U(h)} r) \le r_w\} \quad \text{(S13)}$$

Let $w(h)$ denote the inner minimum,

$$w(h) = \min r = \min \sum_i \{(\sum_L (\sum_j \text{ceil}(\frac{\log(\beta^{\frac{1}{(1-\sigma_i^j F_i^j) C_i (1/R^j)}})}{K \log(1-\sigma_i^j F_i^j)} \times \frac{A_L^j}{A_t^j}))) \times C_i\} \le r_w \quad \text{(S14)}$$

Opposite to this robustness function referred to above in Supplementary Section S1, K, σ, F should be maximum and R be minimum, shown as follows:

$$\sigma_i^j = ((1+h)\tilde{\sigma}_i^j)^+ \quad \text{(S15)}$$

$$F_i^j = ((1+h)\tilde{F}_i^j)^+ \quad \text{(S16)}$$

$$R^j = (1-h)^+ \tilde{R}^j \quad \text{(S17)}$$

$$K = (1+h)\tilde{K} \quad \text{(S18)}$$

Therefore,

$$w(h) = \min r = \sum_i \{(\sum_L (\sum_j \text{ceil}(\frac{\log(\beta^{\frac{1}{[1-((1+h)\tilde{\sigma}_i^j)^+((1+h)\tilde{F}_i^j)^+]C_i[1/((1-h)^+\tilde{R}^j)]}})}{(1+h)\tilde{K}\log[1-((1+h)\tilde{\sigma}_i^j)^+((1+h)\tilde{F}_i^j)^+]} \times \frac{A_L^j}{A_t^j}))) \times C_i\} \le r_w$$

(S19)

As can be seen in the opportuneness function, with the increase of h, the range of uncertainty increases, and the $\min r$ decreases. Thus $w(h)$ decreases as h increases. $\hat{\beta}(r_w)$ is the least value of h at which $w(h) \le r_w$. A plot of h vs $w(h) \le r_w$ is identical to that of $\hat{\beta}(r_w)$ vs r_w. Thus $w(h)$ is the inverse function of $\hat{\beta}(r_w)$. Plots of opportuneness curves in the body of the paper are based on the expressions for $w(h)$.

Table S1. Relative importance weights (RIWs) for entry points for an Asian House Gecko incursion on Barrow Island. Data was based on expert elicitation that was coordinated by Chevron Australia

Points of entry	Description	RIWs
MOF	Material Offloading Facility, where material is offloaded from vessels	0.7
Airport	BWI airport, where airplanes land and passengers transit to BWI accommodation	0.1
Old Airport	Old airplane landing strip that is now used for temporary laydown of cargo and part of which is a waste treatment plant	0.13
Accommodation	Two accommodation facilities on BWI; (1) Butler Park and (2) Production Village. These have been grouped together as a single entry site	0.01
POF	Permanent Operating Facility, which is the main operating offices for the gas treatment plant	0.03
Gorgon LNG Plant	Gorgon Liquefied Natural Gas Plant	0.03
Total		1

Table S2. Relative importance weights (RIWs) for establishment of the Asian House Gecko on Barrow Island[1]

Attributes of habitat suitability	Description	RIWs
Lights	Any free standing lights or lights along roads	0.24
Human Vertical shelters	Man-made structures, buildings	0.65
Natural Vertical Shelters	Trees (coastal acacia), rocky areas	0.11
Total		1

Table S3. Surveillance area of each location on Barrow Island. Data was provided by Chevron Australia.

Surface area (m^2)	Accommodation	POF	Airport	Old airport	Gorgon LNG Plant	MOF	Total
Z1[a]	250,308	60,735	5,011	7,959	55,713	19,826	399,552
Z2	422,191	90,812	72,701	136,467	698,178	0	1,420,348
Z0	0	0	0	0	0	300,000	300,000

[a] The surface area of Z1 does not refer to the entire Zone 1 area on the quarantine invasion risk map (Fig. 1), but only the specified habitat where AHG would be detected (i.e. building area).

Table S4. Definition of Surveillance System Components (SSCs). Data was provided by Chevron Australia.

**Surveillance System Components (SSCs)**	**Description**
Non-networked Environmental Acoustic Recognition Sensors (EARS)	A device that can detect the multiple chirp calls of *Hemidactylus frenatus*. This device requires manual downloading of calls and timestamp of calls.<br><br>One SSC equals 1 EAR recording for 1 night.
Networked EARS	A networked device that can detect the multiple chirp calls of *Hemidactylus frenatus*. This device is powered by solar panels and provides notifications to an end user via a user interface when a suspect call is detected.<br><br>One SSC equals 1 EAR recording for 1 night.
Gecko scat collections	Search of a likely habitat area for signs of an introduced gecko. Areas searched include vertical surfaces near lights. DNA from scat samples is sequenced to determine species identity (see [2]). Sequences are compared to taxa from the international sequence database (GenBank

	(ncbi.nlm.nih.gov/genbank/)), the Helix vertebrate database and the Barrow Island specific database for *Gekkonidae* and *Scincidae* species.<br><br>One SSC equals a 100 m^2 area searched for gecko scats or a 1 hr long search.
Biologist structured surveys	A formal biological survey of an area looking for signs of non-indigenous vertebrate or invertebrate species. Signs may include tracks, scats, auditory calls, burrows, eye shine, eggs or individuals.<br><br>One SSC is approximately 5,000 m^2 area.
Biologist unstructured surveys	The biologist undertaking surveillance on Barrow Island may detect a potential NIS in his/her personal space when travelling around the island, but when not performing a biologist structured survey.
Passive workers [a]	Members of the island workforce who have not had any formal training to detect NIS (aside from the induction), but may notice an unusual vertebrate or invertebrate in their personal work or recreation area and hand it into quarantine.<br><br>One SSC is equivalent to 1 personnel's work on site at any time on Barrow Island during 1 year.

[a] The maximum number of passive workers is 1,000. This number could vary depending on the activities being undertaken on the island.

Table S5. σ, footprint and unit cost of various Surveillance System Components (SSCs) at different locations and zones. Data was based on expert elicitation that was coordinated by Chevron Australia.

**Surveillance System Components (SSCs)**	**Sigma Z1 [a]**	**Sigma Z2**	**Sigma Z0 ***	**Footprint [b] (m^2)**	**Cost [c] (AU$)**
EARS (non-networked)	0.4	0.4	0.2	300	5.5
EARS (networked)	0.45	0.45	0.45	300	2.3
Gecko scat collections	0.74	0.25	0.02	100	110
Biologist structured surveys	0.6	0.2	0.07	5000	110
Biologist unstructured surveys	0.3	0.01	0.035	3000	20
Passive workers	0.05	0	0.01	10	1.1

* Z0 only occurs at the MOF. [a] Sigma is the detection probability of Surveillance System Components given invasive species present in the footprint. [b] Footprint is the area in which an AHG can be detected with a single unit of SSC. [c] Cost is per unit of Surveillance System Components.

References

1. Wintle, B. & Burgman, M. *Expert Elicitation for Barrow Island Surveillance System Revision, Project Report.* (2015).
2. Thomas, M. L. *et al.* Many eyes on the ground: citizen science is an effective early detection tool for biosecurity. *Biol. Invasions* **19**, 2751-2765 (2017).
